# Correlation between functional outcome and the SAMEO-ATO framework

**DOI:** 10.1007/s00405-021-07000-3

**Published:** 2021-07-26

**Authors:** Vito Pontillo, Marialessia Damiani, Giusi Graziano, Nicola Quaranta

**Affiliations:** 1 Otolaryngology Unit, Department of BMS, Neuroscience and Sensory Organs, University of Bari, Bari, Italy; 2 Biomedical Sciences and Human Oncology Department, University of Bari, Bari, Italy; 3 UOC Otorinolaringoiatria Universitaria, Azienda Ospedaliero-Universitaria Consorziale Policlinico Di Bari, Piazza Giulio Cesare 11, 70124 Bari, Italy

**Keywords:** SAMEO-ATO, Hearing, Cholesteatoma, Surgery, Tympanoplasty

## Abstract

**Purpose:**

To evaluate the recently proposed SAMEO-ATO framework for middle ear and mastoid surgery, by correlating it with the functional outcome in a large cohort of patients operated for middle ear and mastoid cholesteatoma in a tertiary referral center.

**Methods:**

We retrospectively included all surgeries for middle ear and mastoid cholesteatoma undergone in our Department between January 2009 and December 2014, by excluding revision surgeries, congenital and petrous bone cholesteatoma. All surgeries were classified according to the SAMEO-ATO framework. The post-operative air bone gap (ABG) was calculated and chosen as benchmark parameter for the correlation analysis.

**Results:**

282 consecutive surgeries for middle ear and mastoid cholesteatoma were released in the study period on a total of 273 patients, with a mean age of 41.2 years. All patients were followed for an average period of 55.3 months. 54% of patients underwent M2c mastoidectomy (Canal Wall Down, CWD), while the remaining underwent Canal Wall Up (CWU) procedures, being M1b2a mastoidectomy the most common one (33%). Mean pre-operative and post-operative ABGs were 29.2 and 23.5 dB, with a significant improvement (*p* < 0.0001). ‘Mastoidectomy’ and ‘Ossicular reconstruction’ parameters of SAMEO-ATO showed significant association with postoperative ABG, with smaller residual gaps for the classes Mx and On, and worse hearing results for M3a and Ox.

**Conclusion:**

Our results show the utility of SAMEO-ATO framework, and in particular of ‘M’ (Mastoidectomy) and ‘O’ (Ossicular reconstruction) parameters, in predicting the hearing outcome.

## Introduction

Cholesteatoma is a mass of keratinizing squamous epithelium located in the middle ear and mastoid that requires surgical treatment. To facilitate the comparison of surgical outcomes and identify prognostic factors, the European Academy of Neurotology and the Japan Otological Society have recently published a joint consensus statement on the definition, classification and staging of middle ear and mastoid cholesteatoma [1]. Other classification systems have been since proposed, such as the “STAMCO” [2] and the “ChOLE” [3]. All the proposed systems describe in detail the characteristics of the cholesteatoma, but none of them takes into account the surgical technique used for its removal.

Surgical treatment of middle ear and mastoid cholesteatoma has been traditionally classified into Canal Wall Up (CWU) and Canal Wall Down (CWD) tympanoplasty [4, 5]; however, the advent of some technical refinements, such as mastoid obliteration [6, 7] and endoscopic ear surgery (EES) [8], has increased the number of surgical techniques and the variables that can affect the surgical outcome.

In 2018, the International Otology Outcome Group (IOOG) proposed a framework that aimed at the categorization of tympano-mastoid surgery [9], with the purpose of favoring the pooling of surgical data in a single large database. The SAMEO-ATO framework categorizes both mastoid and middle ear surgery. The acronym SAMEO stands for Stage of Surgery, Approach, Mastoidectomy, External ear canal reconstruction and Obliteration of the mastoid cavity; the acronym ATO refers to Access to middle ear, Tympanic membrane and Ossicular chain surgical management. In the www.ioog.net website the complete framework is available with clear diagrams and explanation. Recently, ten Tije et al. [10] assessed the value of the SAMEO-ATO classification in the description of the surgery in a multi-centric Dutch cohort and concluded that “the newly proposed classification seems to be more detailed in the registration of surgical procedures that surgeons are currently used to”. One of the theoretical advantages of such classification is that it may allow a more precise comparison of surgical outcomes and may help to identify factors that affect the outcome.

The aim of the present study was to assess the value of the SAMEO-ATO classification by retrospectively correlating its stages with the hearing outcome in a group of patients surgically treated for middle ear cholesteatoma in a tertiary referral center.

## Materials and methods

The study group includes 273 patients affected by middle ear and mastoid cholesteatoma and operated on in our Department between January 2009 and December 2014. All patients that had undergone previous surgery or affected by congenital or petrous bone cholesteatoma were excluded. Nine subjects underwent bilateral intervention; therefore, the total number of surgeries was 282.

Mean age at surgery was 41.2 years (range 3–82 years), 152 (55.6%) were males and 121 (44.3%) females. 146 surgeries were performed on the left side and 136 on the right side. Average post-operative follow-up was 55.3 ± 33.5 months (range 6–136 months).

Demographic data, intra-operative findings, surgical technique, post-operative anatomical and functional findings were all recorded in an electronic database.

The intra-operative extension of cholesteatoma and the presence of complications were evaluated according to two staging systems, EAONO/JOS [1] and STAMCO [2]. Furthermore, the analysis of the operative notes allowed the retrospective classification of the surgical technique according to the SAMEO-ATO framework [9]. In the analysis of hearing results, M3a mastoidectomy was excluded, while M2b and M2c mastoidectomies were considered as CWD procedures and all the remaining cases were considered as CWU tympanoplasties. The guidelines of the Committee on Hearing and Equilibrium of the American Academy of Otolaryngology Head and Neck Surgery [11] were followed and the pure-tone average (PTA) was calculated as the mean of 0.5, 1, 2 and 4 kHz thresholds. Air–Bone Gaps (ABG) were calculated from air conduction (AC) and bone conduction (BC) thresholds. In the evaluation of functional outcome, the postoperative ABG was chosen as benchmark parameter and the surgical program was considered ‘incomplete’ in patients who refused the planned second-stage surgery. All patients signed an informed consent, and the work was performed in accordance with the principles of the 1983 Declaration of Helsinki. Approval was obtained by the local EC.

### Statistical analysis

Categorical or dichotomous variables were expressed as absolute number and percentage (*N*, %). Continuous variables were expressed as mean ± standard deviation (μ ± SD). Chi-square or Fisher’s exact tests and Mann–Whitney test were used to compare categorical and continuous variables, respectively. Wilcoxon test was used to assess the post-operative changes in terms of ABG. A *p* value < 0.05 was considered statistically significant. The software R (version 3.5.2) was used for statistical analysis.

## Results

Table 1 reports the classification of the cholesteatomas according to the EAONO-JOS and STAMCO classifications; in all tables, data for the whole group as well as for the CWU and CWD tympanoplasty groups are reported. According to the EAONO/JOS classification, most of the patients were in stage 2 (cholesteatoma involving two or more sites). Statistical analysis showed that patients in stage 3 (cholesteatoma with extracranial complications) and 4 (cholesteatoma with intracranial complications) were preferentially treated by CWD procedures (*p* < 0.001).

**Table 1:** Distribution of stages according to the EAONO-JOS and STAMCO cholesteatoma classifications in the total population and in CWU or CWD groups

	Stage	Tot. population *N* (%)	CWU *N* (%)	CWD *N* (%)
**EAONO/JOS**	**1**	51 (18.1)	35 (27.3)	16 (10.4)
	**2**	197 (69.9)	88 (68.8)	109 (70.8)
	**3**	32 (11.3)	5 (3.9)	27 (17.5)
	**4**	2 (0.7)	0 (0)	2 (1.3)
**STAM**	**1**	50 (17.7)	33 (25.8)	17 (11)
	**2**	56 (19.9)	23 (18)	33 (21.5)
	**3**	176 (62.4)	72 (56.2)	104 (67.5)
**C**	***n***	248 (87.9)	123 (96.1)	125 (81.2)
	**1**	32 (11.3)	5 (3.9)	27 (17.5)
	**2**	2 (0.8)	5 (3.9)	27 (17.5)
**O**	***n***	51 (18.01)	26 (20.3)	25 (16.2)
	**1**	111 (39.4)	53 (41.4)	58 (37.7)
	**2**	81 (28.7)	41 (32)	40 (26)
	**3**	39 (13.8)	8 (6.3)	3 (20.1)

Bold values: statistically significant results (p-value<0.005)

According to the STAMCO classification, two-thirds of patients were classed as STAM 3 (cholesteatoma in three locations or in one difficult site). Statistical analysis showed that CWD procedures were significantly more frequent in more extensive cholesteatomas (higher STAM stages) (*p* = 0.0055), in case of complications (*p* = 0.002) and greater ossicular chain involvement (*p* = 0.0092).

In Table 2, the classification of the surgeries according to the SAMEO/ATO framework is reported. Almost 70% of interventions were staged, and the approach was retro-auricular in 90% of cases. 54% of patients underwent an M2c mastoidectomy, while the remaining 45% underwent CWU procedures, being a M1b2a mastoidectomy the most common one (33%). The ear canal was never reconstructed entirely, while in case of M2a mastoidectomy, the scutum was usually reconstructed with cartilage or bone paste. In M2c, the mastoid was partially obliterated with bone paste in most of the cases (131/152).

**Table 2:** Surgery classification according to the SAMEO/ATO framework

Category	Class	*N* (%)	Category	Class	*N* (%)
**S** (Stage of surgery)	**S1**	90 (31.9)	**A** (Access to the middle ear)	**Ax**	267 (94.7)
	**S2**	192 (68.1)		**A1**	5 (1.8)
**A** (Approach)	**A1**	0		**A2**	10 (3.5)
	**A2**	29 (10.3)		**A3**	0
	**A3**	0	**T** (Tympanic membrane)	**Tx**	2 (0.7)
	**A4**	253 (89.7)		**Tn**	0
**M** (Mastoidectomy)	**Mx**	14 (5.0)		**T1**	4 (1.4)
	**M1a**	0		**T2**	32 (11.3)
	**M1b**	0		**T3**	244 (86.6)
	**M2a**	15 (5.3)	**O** (Ossicular chain)	**Ox**	69 (24.5)
	**M2b**	0		**On**	23 (8.2)
	**M2c**	152 (53.9)		**Osi**	5 (1.8)
	**M1a + 2a**	6 (2.1)		**Osm**	0
	**M1b + 2a**	93 (33.0)		**Ost**	87 (30.9)
	**M3a**	2 (0.7)		**Osd**	26 (9.2)
	**M3b**	0		**Ofi**	0
**E** (Ear canal reconstruction)	**Ex**	168 (59.6)		**Ofm**	0
	**E1**	0		**Oft**	68 (24.1)
	**E2**	114 (40.4)		**Ofd**	4 (1.4)
**O** (Obliteration)	**Ox**	150 (53.2)		**Ovi**	0
	**O1**	101 (35.8)		**Ovm**	0
	**O2**	31 (11.0)		**Ovt**	0

Bold values: statistically significant results (p-value<0.005)

Table 2 reports the classification of surgeries according to the ATO framework. For the reconstruction of the ossicular chain, homologous costal cartilage was used in 51.6% of cases, autologous ossicles in 25.8%, tragal cartilage in 15.1%, titanium prosthesis in 6.9% and bone paste in 0.6%.

In Table 3, hearing results in terms of AC PTA and ABG are reported for the pre- and post-operative periods for the whole population and for the CWU and CWD groups. In the entire sample, a significant improvement in the post-operative period was found both for AC-PTA and ABG, while average BC did not change significantly. In the post-operative period, almost 50% of patients showed an ABG ≤ 20 dB.

**Table 3:** Functional results in the whole population and in the CWU or CWD groups

	Total	CWU	CWD	*p* value (CWU vs CWD)
Delta-BC PTA (µ ± DS)	− 2.4 ± 11.5	− 0.8 ± 7.1	− 3.8 ± 14.2	0.1303
Pre-op PTA_AC_ (µ ± DS)	52.3 ± 20.2	50.2 ± 18.9	54.1 ± 21.2	0.2229
Post-op PTA_AC_ (µ ± DS)	48.6 ± 23.0	42.6 ± 19.5	54.4 ± 24.7	**0.0005**
Last PTA_AC_ (µ ± DS)	50.2 ± 21.9	47.8 ± 20.5	52.8 ± 23.1	0.236
***p* value (pre- vs post-op)**	**0.0012**	** < 0.0001**	0.4687	
***p* value (post-op vs last)**	** < 0.0001**	** < 0.0001**	0.054	
Pre-op ABG (µ ± DS)	29.2 ± 12.1	29.0 ± 12.4	29.4 ± 12.0	0.6265
Post-op ABG (µ ± DS)	23.5 ± 13.7	20.8 ± 12.0	26.2 ± 14.7	**0.0054**
Last ABG (µ ± DS)	25.3 ± 13.7	25.8 ± 14.2	24.7 ± 13.2	0.704
***p* value (pre- vs post-op)**	** < 0.0001**	** < 0.0001**	**0.0053**	
***p* value (post-op vs last)**	**0.002**	**0.001**	0.49	
Post-op air bone gap				
ABG ≤ 20 dB *n* (%)	98 (46.7)	57 (54.8)	41 (38.7)	**0.019**
ABG > 20 dB *n* (%)	112 (53.3)	47 (45.2)	65 (61.3)	

Bold values: statistically significant results (p-value<0.005)

In the CWU group, both AC-PTA and ABG improved after surgery, while in the CWD group, only ABG improved. Moreover, significant better post-operative AC-PTA and ABG were found in the CWU group compared to CWD. 54.8% and 38.4% of patients presented a post-operative ABG ≤ 20 dB in the CWU and CWD groups, respectively, being the significant difference (*p* = 0.019).

As reported in Table 3, long-term pure-tone audiometry, recorded at an average time of 4.3 ± 2.6 years from surgery (min 2, max 10 years), showed a deterioration of AC-PTA (*p* < 0.0001) and ABG (*p* = 0.001) in the CWU group, while results were more stable in the CWD group.

Residual cholesteatoma was found to be significantly more frequent in the CWU group (17.2 vs 3.9%, *p* = 0.0002 of CWD), as well as recurrent cholesteatoma (7 vs 0% of CWD, *p* = 0.001). Other long-term anatomical complications, such as TM retraction, granulations or epidermoid cysts, were found in 8.6% of patients submitted to a CWU and in 7.1% of those who had undergone a CWD procedure, with no significant difference between the two groups (*p* = 0.652). Long-term functional complications, such as dislocation, extrusion or absorption of the ossiculoplasty material, were recorded in 8 cases of CWU (6.25%) and in 2 cases of CWD (1.3%), with only a trend towards significance between the two techniques (*p* = 0.047).

Table 4 shows the results of the univariate correlation analysis between post-operative ABG and the pre- and intra-operative factors for the entire population and CWU and CWD groups.

**Table 4:** Univariate correlation analysis between post-operative ABG and pre-operative or intra-operative features

	Total population		CWU		CWD	
	Postop ABG µ ± DS	*p* value	Postop ABG µ ± DS	*p* value	Postop ABG µ ± DS	*p* value
Cholesteatoma stages						
EAONO/JOS						
1	20.7 ± 14.3	0.1685	20.9 ± 12.3	0.7686	19.9 ± 20.8	0.1416
2	23.7 ± 12.6		20.5 ± 11.9		26.6 ± 12.5	
3	26.5 ± 19		25.8 ± 14.5		26.6 ± 20.1	
4	41.3 ± n.d		n.d		41.3 ± nd	
STAM						
1	20.5	0.1694	20.4	0.7131	20.4	0.155
2	23.9		18.8		26.9	
3	24.2		21.4		26.7	
Complications						
*N*	23.1 ± 13.0	0.3212	20.6 ± 12.0	0.4796	25.9 ± 13.6	0.5284
1	26.5 ± 19.0		25.8 ± 14.5		26.6 ± 20.1	
2	41.3 ± n.d		n.d		41.3 ± n.d	
Ossicular chain status						
*n*	19.1 ± 12.1	**0.0038**	18.0 ± 12.1	0.1685	20.5 ± 12.3	**0.0677**
1	22.1 ± 13.1		19.4 ± 10.3		25.0 ± 15.2	
2	25.9 ± 12.5		23.8 ± 12.4		28.3 ± 12.4	
3	29.7 ± 17.6		25.0 ± 18.7		31.2 ± 17.4	
Surgical program						
Complete	22.2 ± 13.1	**0.0016**	20.5 ± 12.3	0.2463	24.1 ± 13.9	**0.0144**
Incomplete	31.0 ± 14.4		23.8 ± 7.8		33.5 ± 15.4	
SAMEO-ATO						
S1	21.6 ± 15.3	0.0797	16.0 ± 13.0	**0.0248**	24.5 ± 15.8	0.1948
S2	24.3 ± 12.9		22.0 ± 11.5		27.3 ± 14.0	
Mx	17.9 ± 11.5	**0.016**	17.9 ± 11.5	0.1823	–	0.112
M2a	15.8 ± 12.2		15.8 ± 12.2		–	
M2c	25.9 ± 14.6		–		25.9 ± 14.6	
M1a + 2a	25.6 ± 9.7		25.6 ± 9.7		–	
M1b + 2a	21.7 ± 12.0		21.7 ± 12.0		–	
M3a	56.3 ± n.d		–		56.3 ± n.d	
Ox	33.7 ± 15.8	** < 0.0001**	26.0 ± 9.9	**0.0454**	36.3 ± 16.7	**0.0006**
On	13.1 ± 9.4		14.0 ± 12.9		12.3 ± 5.9	
Osi	19.3 ± 10.1		12.1 ± 2.9		30.0 ± 1.8	
Ost	21.3 ± 11.2		20.1 ± 10.7		23.3 ± 12.1	
Osd	24.8 ± 13.3		n.d		24.8 ± 13.3	
Oft	23.5 ± 13.2		22.6 ± 13.5		25.1 ± 12.9	
Ofd	24.4 ± 11.5		n.d		24.4 ± 11.5	
Ossiculoplasty material						
Bone paté	8.8 ± n.d	0.1445	8.8 ± n.d	0.3194	–	0.4516
Costal cartilage	22.3 ± 13.4		21.7 ± 12.8		23.9 ± 15.1	
Autologous ossicles	19.6 ± 11.4		19.4 ± 10.8		20.1 ± 13.8	
Titanium	23.3 ± 8.0		18.3 ± 4.6		31.7 ± 3.8	
Tragal cartilage	25.8 ± 8.7		31.9 ± 8.0		25.1 ± 8.7	

Bold values: statistically significant results (p-value<0.005)

### Study group

None of the cholesteatoma staging systems, except the ‘ossicular chain status’ at the beginning of surgery of the STAMCO classification, correlated with post-operative hearing outcome; in fact, higher ‘O’ stages were associated with significantly poorer hearing results for the whole population (*p* = 0.0038). Incomplete surgical program negatively correlated with the functional outcome (*p* = 0.0016). In regard to the SAMEO-ATO framework, the Approach, External ear reconstruction, Obliteration, Access to middle ear and Tympanic membrane reconstruction were not evaluated in the statistical analysis, since most of the patients were staged as E2, O1, Ax and T3.

Mx and M2a classes showed the best hearing results in terms of postoperative ABG, while the worst results concerned the patients submitted to M3c mastoidectomies (*p* = 0.016). CWU tympanoplasties as a group (Mx, M2a, M1a + 2a and M1b + 2a) were associated with better post-operative hearing function both in terms of average ABG and the number of patients with an ABG better than 20 dB HL. Comparing the most frequently used mastoidectomy techniques, M1b + 2a showed better post-operative ABG averages than M2c (21.7 ± 12 vs 25.9 ± 14.5 dB HL; *p* = 0.034).

In terms of ‘ossicular reconstruction’ (O), On (intact chain preservation) and Osi (reconstruction between incus and stapes head in case of limited erosion of the long process of the incus) classes were associated with the lowest ABG (respectively, 13.1 and 19.3 dB), while the worst results were registered in cases where no reconstruction was performed (Ox, mean ABG = 33.7 dB). No significant differences in terms of post-operative ABG were encountered when comparing partial (Osi, Ost, Osd) with total (Oft, Ofd) ossiculoplasty or the materials used for reconstruction. However, long-term post-operative ABG showed significant better values when autologous ossicles had been used for reconstruction (19.2 ± 13.8 dB) compared to allogenic costal cartilage (27.9 ± 14.2 dB; *p* = 0.008) and titanium prosthesis (32.7 ± 13.4 dB; *p* = 0.028).

A within-groups comparison showed that in CWUT single-stage surgery, intact ossicular chain at the end of surgery (On) and minimal reconstruction (Osi) were all associated with better hearing results. In CWDT, incomplete surgery was a negative prognostic factor, while an intact chain at the end of surgery was associated with better hearing results (Table 4).

## Discussion

In the present study, the SAMEO-ATO classification was correlated with the functional outcome in a large cohort of patients affected by middle ear and mastoid cholesteatoma.

The SAMEO-ATO framework has been recently proposed to categorize the surgical techniques used for the treatment of cholesteatoma and allow the comparison of surgical results [9]. The role of cholesteatoma staging systems in predicting surgical outcome has been reported by different authors. Van der Tom et al. [12] evaluated the role of the EAONO/JOS [1] and STAMCO [2] classifications and demonstrated STAMCO’s superiority in predicting both residual and recurrent disease. However, this finding was only referred to patients treated with CWU tympanoplasty, since recurrent and residual cholesteatoma rates were negligible in the CWD group. In terms of hearing results, the authors found, in the CWU group, larger post-operative ABG with increasing stage (both EAONO/JOS and STAM), complications and ossicular chain status; on the contrary, in the CWD group, increasing ossicular chain status correlated with higher AC PTA, with no effect on ABG. Angeli et al. [13] evaluated the prognostic value of the EAONO/JOS classification in terms of recidivism, in a group of patients affected by cholesteatoma and treated with a CWU tympanoplasty. The authors showed that the recidivism rate was higher in children, in case of larger bone canal defect or cholesteatoma located in the supra-tubal recess; however, the prognostic role of the classification was defined as “uncertain” [13]. Ardıç et al. [14] correlated the surgical outcomes with the EAONO/JOS staging system and reported no correlation between the staging system and the recurrence rate. Although they described the number of patients treated with CWU and CWD procedures, the authors did not analyze the role of surgical technique in the recidivism rate. Fukuda et al. [15] reported the short-term hearing results in a small group of patients affected by pars flaccida cholesteatoma and submitted to mastoidectomy and tympanoplasty with cartilage double-block reconstruction on the stapes. The authors classified all the cases with the EAONO/JOS staging system and used a post-operative ABG < 20 dB as success criterion, thereby finding that poorer hearing results were associated with higher EAONO/JOS stages and stapes involvement.

It is therefore clear from the existing literature that the pre-operative stage does not consistently predict by itself the post-operative outcome, especially when the surgical technique used to treat cholesteatoma is not taken in consideration.

In the present study, we have correlated the post-operative hearing results both with cholesteatoma staging systems and surgical procedure. In particular, this is the first study that evaluates the correlation of the SAMEO-ATO framework with the hearing outcome. Statistical analysis showed that, in regard to cholesteatoma staging systems, the main factors responsible for the post-operative hearing are a complete surgical program, the status of the ossicular chain at the beginning of surgery and the surgical technique used.

van der Toom et al. [12], using the STAMCO classification, showed that the ossicular chain status at the beginning of surgery was the only factor correlating with hearing outcome.

Our correlation analysis of the SAMEO framework showed that the M status associated with hearing results; within the ATO framework, the O status showed its value, in particular an intact ossicular chain at the end of the procedure (On) was associated with the best hearing results.

As a group, CWU procedures were associated with better post-operative hearing in terms of AC PTA and ABG compared to CWD procedures. Although these results could be easily explicable in case of no (Mx) or minimal mastoid surgery (M2a) since related to the small size of the cholesteatoma and to the minimal involvement of the ossicular chain, it should be highlighted the fact that even the comparison between M1b2a (mastoidectomy with posterior tympanotomy and scutum reconstruction) and M2c (canal wall down mastoidectomy with partial obliteration) confirmed these findings in our analysis. Similar results were reported by other authors that compared CWU and CWD procedures [16–19]. The presence of a larger middle ear space and a near-normal anatomy allows a more “physiological” reconstruction of the ossicular chain.

As reported in a recent meta-analysis [20], also in the present series, CWUT was associated with significantly higher residual and recurrent cholesteatoma; therefore, the choice between CWUT and CWDT procedures should be balanced also considering the higher risk of recurrence in “closed” procedures.

The preservation of the ossicular chain both in CWU and CWD tympanoplasties was associated with the best hearing results. In case of an intact ossicular chain at the beginning of the procedure, it can be saved using both CWU and CWD techniques. Our group has showed that in case of cholesteatoma located only in the middle ear space, the preservation of the ossicular chain is possible maintaining the posterior wall of the EAC intact, while in case of epitympanic cholesteatoma with extension in the mastoid antrum, the Bondi technique allows the preservation of hearing with minimal recurrence rates [21]. Similar results have been reported by other authors [22–24].

In terms of reconstruction of the ossicular chain, no significant differences were encountered in terms of partial or total reconstruction. Yu et al. [25] performed a meta-analysis of the existing literature and compared the effect of PORPs and TORPs for the ossicular chain reconstruction. PORPs were comprehensively more effective than TORPs in terms of hearing function, but no difference was detected in staged procedures subgroup and in cholesteatoma subgroup, similarly to the present series where most of the surgeries were staged and all the patients were affected by cholesteatoma.

## Conclusion

Cholesteatoma classification and staging systems have gained increased attention in recent years. In particular, EAONO/JOS and STAMCO classifications have demonstrated their utility in various aspects of clinical evaluation, but they mostly failed in terms of predictive power both in terms of cholesteatoma recidivism and hearing outcome. This is likely attributable to the fact that none of these classification systems considers the specific surgical features. The SAMEO-ATO framework has been recently proposed to categorize the surgical techniques used for the treatment of cholesteatoma and allows a more direct comparison of surgical results. To our knowledge, this is the first study evaluating the correlation of the SAMEO-ATO framework with the hearing outcome. Our results show the utility of this framework, and in particular of ‘M’ (Mastoidectomy) and ‘O’ (Ossicular reconstruction) parameters, in predicting hearing outcome. Besides, the analysis of our series points up the need of an integrated classification system that combines both pathological and surgical evaluations.

## Data Availability

All data and material are available and can be provided if requested.
